# A Simple and Efficient Route for Preparing 2,3,5,6-Tetraaminopyridine Hydrochloride Salt

**DOI:** 10.3390/molecules14051652

**Published:** 2009-04-27

**Authors:** Yanhong Wang, Zhen Hu, Xiangli Meng, Jiehui Jing, Yuanjun Song, Chunhua Zhang, Yudong Huang

**Affiliations:** Department of Applied Chemistry, Faculty of Science, Harbin Institute of Technology, PO Box 410#, Harbin 150001, P. R. China

**Keywords:** 2,3,5,6-Tetraaminopyridine, Nitration, Hydrogenation, Synthesis.

## Abstract

A simple and efficient route for preparing 2,3,5,6-tetraaminopyridine hydrochloride salt (TAP·3HCl·H_2_O) was introduced in this paper. The title compound was synthesized, as usual, in two steps (nitration and hydrogenation) with a total yield of 90%. The use of an oleum and fuming nitric acid mixture in the nitration step improved the yield and purity of the intermediate product. A highly efficient hydrogenation using a H_2_/Pd/C/C_2_H_5_OH system was developed. The products were characterized by TG, IR, ^1^H-NMR, ^13^C-NMR, HPLC and elemental analysis.

## Introduction

In the last two decades, explosive progress has been made in synthetic methods for production of aromatic and heterocyclic amino compounds, due to their rapidly increasing applications in pharmaceutical and material sciences. Polymer matrix composites (PMC) that incorporate these types of compounds as monomers may have applications in military and aerospace designs such as aircraft fuselages, naval ship hulls, and body armor due to their light weight and strength properties [1,2]. An important monomer used in the preparation of such high-performance polymers is 2,3,5,6-tetraamino-pyridine (TAP), whose extreme sensitivity to air oxidation makes its isolation and purification challenging and can lead to polymers with suboptimal properties. Due to TAP’s instability, it is typically isolated as its hydrochloride salt [3].

Nitration reactions using conventional electrophilic conditions are highly disfavored in the heteroaromatic series due to the presence of the heteroatom, which interacts with the electrophile to give onium species [4]. Furthermore, nitration of 2,6-diaminopyridine(DAP) at a ring carbon atom with nitric acid, nitric acid-sulfuric acid mixtures, or other common nitrating systems generally results in a very low yield of nitropyridine [5,6,7]. The formation of the pyridinium salt deactivates the pyridine ring and prevents further reaction [8]. Besides, the action of a more powerful nitrating agent such as nitronium tetrafluoroborate on aminopyridine leads to the formation of the corresponding *N*-nitropyridinium salt, with no *C*-nitro compound being obtained [9]. It has been reported in the literature [10] that with mixed acid the nitryl base (-NO_2_) migrated to the amino group of 2-aminopyridine first, and then the resulting intermediate rearranged under the strong acid conditions. Our experiments had verified that when the above method is used more byproducts are formed too, so the nature of the nitrating agent plays an important role in the outcome of the nitration of DAP.

A number of methods have been developed for the reduction of nitropyridines [11,12,13,14], however, they often suffer from serious disadvantages, such as incompatibility with other functional groups, low yields, harsh reaction conditions, and difficult work-up procedures. The free amino groups at the 3,5-positions largely account for TAP’s air sensitivity. Attempts to prepare 3,5-diaminopyridine from 3,5-dibromopyridine by the Maier-Bode procedure were unsuccessful [15]. Davis and Irvin synthesized the tetraethylcarbamate of 2,3,5,6-tetraaminopyridine in 10 steps with 23% overall yield using dinicotinic acid as starting material [16]. All the intermediates in this reaction sequence were shelf stable, but the process was not amenable to pilot plant scale-up due to the poor yield and unwanted byproducts. Furthermore, it would be difficult to prepare polypyridobisimidazole (PIPD) from the tetraethylcarbamate of 2,3,5,6-tetraaminopyridine. Sikkema [2] had reported that TAP was synthesized by the reduction of 2,6-diamino-3,5-dinitropyridine (DADNP) in a phosphoric acid/H_2_/catalyst system. The hydrogenation process was complex, time-consuming and not environmentally friendly. The Pd/C catalyst was hard to recover due to the acidic system.

**Scheme 1:**
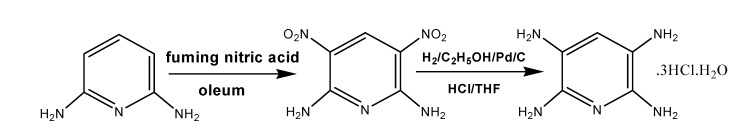
Route for Preparing 2,3,5,6-Tetraaminopyridine Hydrochloride Salt.

With the increasing current interest in environmental protection, more attention is being paid to “Green Chemistry”, so we decided to explore the possibility of finding a clean system to perform the described type of reduction. We report herein a more environmentally friendly and highly efficient method for the hydrogenation of DADNP as shown in Scheme 1. The compound TAP·3HCl·H_2_O was synthesized using DAP as a starting material in two steps (nitration and hydrogenation) with a total yield of above 90%.

## Results and Discussion

**Figure 1:**
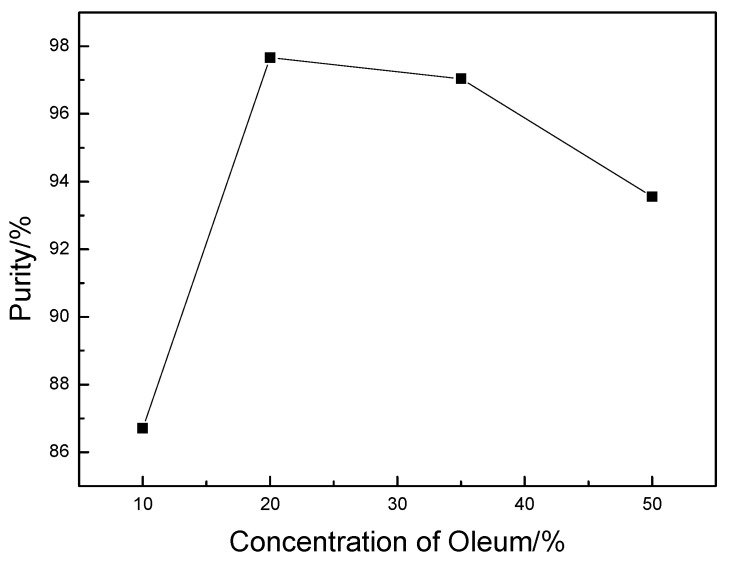
Influence of the oleum concentration(%).

**Figure 2:**
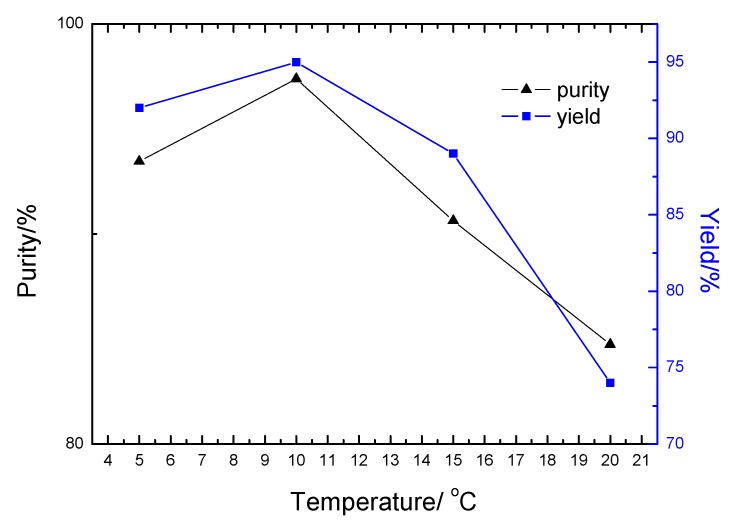
Influence of t_n_ on purity and yield of DADNP.

**Figure 3:**
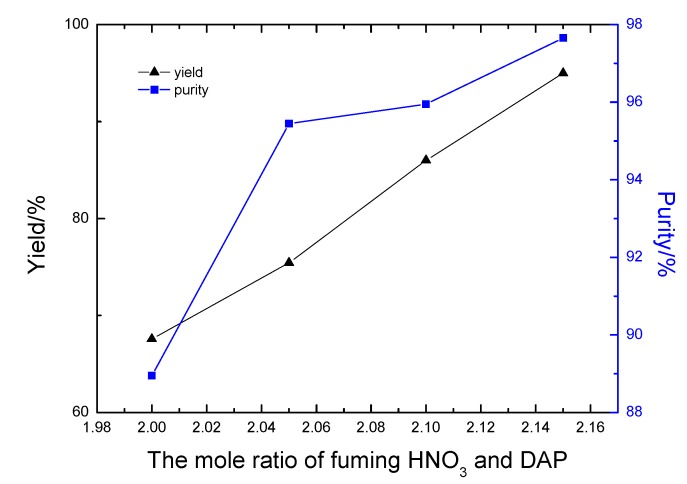
Influence of the mole ratio of fuming nitric acid and DAP on purity and yield of DADNP.

**Figure 4:**
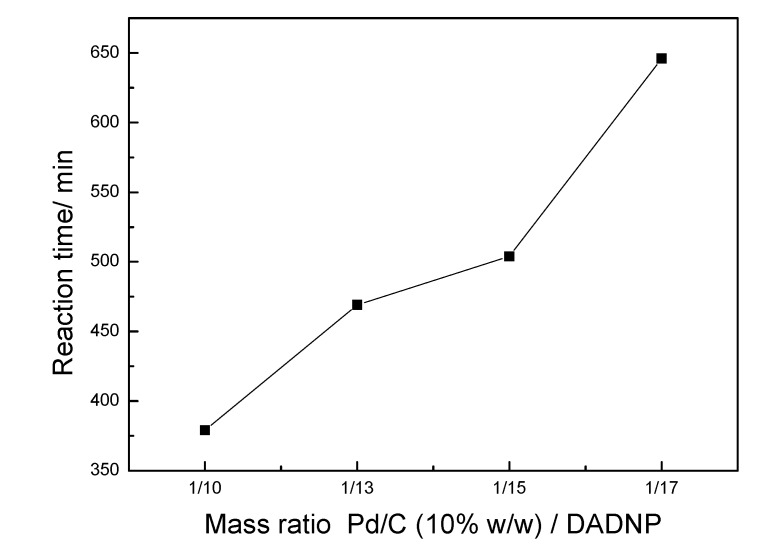
Influence of the mass ratio of Pd/C (10% w/w) and DADNP on the reaction time.

In this work, we have improved the nitration of DAP reported by Sikkema [2,5]. We adopted the direct nitration of DAP using an oleum and fuming nitric acid mixture, in which both the purity and yield of DADNP were greatly improved. Figure 1, Figure 2, Figure 3, Figure 4 show the results of our systematic investigation of the preparation of DADNP and the results obtained, as well as for the hydrogenation of DADNP to TAP, which are essential for understanding the discussion. DAP could participate in β-electrophilic substitution reactions, but the transition state energy will be higher due to electrostatic repulsion, so . its electrophilic substitution reactions would be very difficult to effect. The α-positioned amino groups in DAP (2-, 6-) because of its directing effects, favors formation of the β-substituted (3 - and 5 -) product . There is no tetranitration because of the inter-NO_2_-group directing effect.

We confirmed the key factors that influenced the yield and purity of DADNP through systematical investigation on the preparation of DADNP. Oleum could reduce degradation by hydrolysis of nitration products and give a surprisingly higher yield due to the dehydration and oxygenation of oleum. Different concentrations of oleum (%) was examined for the nitration reaction. Figure 1 summarizes the influence of this factor on the purity of DADNP. The highest purity was up to 97.66% when 20% oleum was used. For industrialization purposes, 20% oleum was the best choice.

Furthermore, the oleum not only promoted the creation of NO_2_^+^, but it also facilitated the carbonization or oxidation of the DAP or DADNP to a certain extent. Thus, higher reaction temperatures, the color of product became dark due to the carbonization or oxidation of DAP. After numerous experiments, it was found that the purity and yield of DADNP was higher in lower nitration temperature (t_n_ <15 °C). Figure 2 shows the influence of the nitration temperature (t_n_) on the purity and yield of DADNP.

The NO_2_^+^ species acts as an electrophilic reagent in the nitration reaction and the presence of protons should facilitate the production of NO_2_^+^, so obviously, the use of fuming nitric acid (85%-100% nitric acid) should improve the nitration step. In addition, the nitration of DAP was a di-nitration reaction, so the molar ratio of fuming nitric acid and DAP should be controlled at 2.0-2.15. As shown in Figure 3, the majority of DADNP purities were in excess of 94%. The purity of DADNP was up to 97.66% when the molar ratio of fuming nitric acid and DAP was 2.15. Furthermore, the yield of DADNP also increased with the molar ratio.

The mode of quenching could influence the purity and yield of DADNP considerably, even though the nitration medium was changed from concentrated sulfuric acid to oleum to avert the production of some by-products. Due to release of uncontrolled heat due to dilution of the acid the elimination of these by-products still depends on the success of the quenching operation. The contact time between the reaction mixture and ice should be kept as short as possible and at the lowest possible temperature, while high-speed and vigorous stirring are maintained, otherwise, a lot of red foamy product is formed engendered that became red-brown after drying. Importantly, optimal synthesis conditions for DADNP were determined by orthogonal experiments. It was found that: the direct nitration of DAP using 20% oleum and 95% fuming nitric acid is a good approach for preparation of DADNP. The purity and yield of DADNP was better when t_n_ was <15°C and the mole ratio of fuming nitric acid and DAP was 2.15:1. The purity of DADNP exceeded 97% according to the HPLC measurement, and the average yield of DADNP was above 95% under the optimal conditions.

The availability of high purity DADNP laid a foundation for the production of high purity TAP. In the early experiments, we investigated the synthesis of TAP by using DADNP and HCOONH_4_-Pd/C, however, the reaction conversion was very low due to a lack of H_2_. It was shown that the hydrogenation process was largely complete under a H_2_ atmosphere (0.8~1.2 MPa), and the hydrogen consumption depended on the quantities of DADNP. Much of the success of hydrogenation depends on the type of catalyst used. Initial small-scale catalytic hydrogenation studies using dry palladium on carbon proved problematic due to the limited solubility of DADNP in several common organic solvents, as well as the difficulty of reclaiming the catalyst. Dilute phosphoric acid was investigated as hydrogenation solvent; however, the substrate was only sparingly soluble in this medium. Dissolution could be achieved by addition of more solvent, or by performing the hydrogenation at higher temperatures [17] as reaction rates were modest at ambient temperature, but the reaction was time-consuming and impurity profiles at elevated temperatures were unacceptable as product isolated by crystallization from hydrochloric acid typically contained 3-4% unidentified impurities. Greater success was achieved when dilute phosphoric acid was replaced by ethanol as reaction solvent. The reaction was complete within 6 h, and the product was readily precipitated from hydrochloric acid. Yields were above 90%, and the impurity profile was considerably improved, compared to material prepared using dilute phosphoric acid. To allow safe scale-up to the pilot plant, the use of wet palladium on carbon was investigated (5% w/w Pd/C containing 50% water). This gave 85% yield, comparable reaction times, and acceptable purities, but required a higher catalyst loading, equivalent to 10% w/w catalyst/substrate. Pd/C (10% w/w) was more amenable to the reduction of NO_2_ groups [18]. When the mass ratio of Pd/C (10% w/w) and DADNP was enhanced to 1:10, full conversion was obtained in short reaction time, as seen in Figure 4.

The catalyst Pd/C could be recycled and reused easily because the active catalytic species was stable under the catalytic conditions used. The hydrogen consumption depended on the quality of DADNP, which avoided higher reaction pressures to a large extent. Attempts to remove the impurities by crystallization with 36% hydrochloric acid showed modest results. This led to a reinvestigation of crystallization from 36% hydrochloric acid/THF mixtures. Superior results were obtained with 36% hydrochloric acid /THF (1:1 v/v), at ambient temperature, which gave optimum impurity removal and a crystalline product. Hence, ethanol was the solvent of choice for hydrogenation and this process improvement was introduced in laboratory use-tests of TAP produced in the pilot-plant, using the optimum process, which gave crystalline product in above 90% yield, 99.75% overall purity and > 99.9% purity after re-crystallization, according to HPLC data. In summary, this hydrogenation procedure was shown to be versatile, effective, and scalable in the laboratory, with the product isolated as a crystalline solid, in high yield.

## Conclusions

We have developed a simple and efficient process for preparing TAP·3HCl·H_2_O using nitration with oleum/fuming HNO_3_ and hydrogenation with an H_2_/Pd/C/C_2_H_5_OH system, which would be amenable to pilot plant scale-up. The nitration medium (oleum and fuming nitric acid) avoids the production of hydrolytic degradation products and increases the yield. The hydrogenation conditions were mild, due to the ethanol medium, and safe, thanks to the low H_2_ pressure. Importantly, this method retains the high reactivity of the catalyst whilst satisfying the need to recover the catalyst. Further investigation on the mechanism and the optimal reaction condition is currently in progress.

## Experimental

### General

All commercially available chemicals and reagents were purchased from Sinopharm Group Chemical Reagent Co., Ltd. and used without further purification. Melting points were determined with an thermogravimetric analysis Q50 apparatus and are uncorrected. IR spectra were recorded on a Nexus 670 instrument. The ^1^H- and ^13^C-NMR spectra were recorded on a Bruker DRX-400 AVANCE spectrometer. Unless otherwise specified DMSO-d_6_ and D_2_O were used as solvents. Chemical shifts (δ) are reported in ppm referenced to the residual NMR solvent peak. The HPLC analysis was performed on an Agilent 1100 system. The purity of the products was measured by HPLC analysis (the areas are calibrated against a previously determined calibration curve, and they are area %). Elemental analyses were obtained on a Vario MAX CHN apparatus.

### Preparation of DADNP

In a 1-liter reactor equipped with a rapid stirrer, 20% oleum (300 mL) was cooled to 0 °C. DAP (60 g) was added slowly in portions, and the temperature had been kept at 20 °C for 2 h. Then 95% fuming nitric acid (52.8 mL) was slowly added while the temperature was kept below 15 °C, and then the reaction mixture was kept at this temperature for 3 h. Finally, the somewhat viscous reaction mixture was stirred into 2 kg of crushed ice, so the final temperature of the mixture was 0 °C. The solids were filtered off, washed three times with ice–water and dried for 24 h at 50 °C to give a yellow solid identified as DADNP (yield 95%, purity 97.67%**)** by its analytical data and comparison with literature values [2]; m.p.: >322 °C; IR (KBr, cm^-1^): 3480, 3363, 3093, 1650(s), 1551, 1508, 1429, 1320, 1239, 857, 785; ^1^H-NMR (DMSO) (ppm): 8.21 (s, 2H, NH_2_), 8.41 (s, 2H, NH_2_), 9.00 (s, 1H, CH); ^13^C-NMR (DMSO) δ: 120.18 (s, 2C, CNO_2_), 135.53 (s, 1C, CH), 154.75 (s, 2C, CNH_2_); Anal. calcd. for C_5_H_5_N_5_O_4_: C 30.15,H 2.51, N 35.17; found C 30.53, H 2.58, N 35.23.

### Preparation of TAP·3HCl·H_2_O

DADNP (50 g), Pd/C (5 g, 10%) and ethanol (350 mL) were hydrogenated under 0.8~1.2 MPa hydrogen pressure in a 1-liter hydrogenation reactor equipped with a rapid stirrer. The reaction proceeded at 50~70 °C for 10 h. After the catalyst was filtered, the filtrate was added to a mixture of 36% HCl and THF (1:1 volume 400 mL), where the light yellow crystalline 2,3,5,6-tetraaminopyridine hydrochloride salt (TAP·3HCl·H_2_O) was formed after about 12 h. Filtration under nitrogen and drying for 24 h at 50 °C yielded TAP·3HCl·H_2_O of 99.75% purity. The identity of the product was established by its analytical data and comparison with the values reported in the literature [2]. IR (KBr, cm^-1^) *ν*: 3360, 3140, 3020, 2800, 2600, 1653, 1606, 1560, 1490, 1385, 1296, 1130, 1100, 1080, 990; ^1^H-NMR (D_2_O) (ppm): *δ*: 7.51 (s, 1H, CH); ^13^C-NMR (DMSO) *δ*: 124.78 (s, 2C, CNH_2_)(C-3,5), 136.73 (s, 1C, CH), 151.36 (s, 2C, CNH_2_)(C-2,6); Anal. calcd. for C_5_H_9_N_5_·3HCl·H_2_O: C 22.53, H 5.29, N 26.27; found C 22.23, H 5.19, N 25.98.
